# Safety and efficacy of remimazolam compared with midazolam during bronchoscopy: a single-center, randomized controlled study

**DOI:** 10.1038/s41598-023-47271-w

**Published:** 2023-11-22

**Authors:** Sun-Hyung Kim, Jun Yeun Cho, Miyeon Kim, Ji Min Chung, Jiyoul Yang, Changhwan Seong, Eung-Gook Kim, Jeong Won Seok, Yoon Mi Shin, Ki Man Lee, Kang Hyeon Choe, Joung-Ho Han, Bumhee Yang

**Affiliations:** 1 Division of Pulmonary and Critical Care Medicine, Department of Internal Medicine, Chungbuk National University Hospital, Chungbuk National University College of Medicine, Cheongju, Korea; 2 Academic Cooperation Foundation, Chungbuk National University Industry, Cheongju, Korea; 3 Red Cross College of Nursing, Chung-Ang University, Seoul, Korea; 4 Department of Clinical Pharmacology & Therapeutics, Chungbuk National University Hospital, Cheongju, Korea; 5 Department of Biochemistry, Chungbuk National University College of Medicine, Cheongju, Korea; 6 Department of Internal Medicine, Chungbuk National University Hospital, Chungbuk National University College of Medicine, Cheongju, Korea; 7 Division of Gastroenterology, Department of Internal Medicine, Chungbuk National University Hospital, Chungbuk National University College of Medicine, Cheongju, Korea

**Keywords:** Drug safety, Target identification, Randomized controlled trials

## Abstract

Although remimazolam is an ultra-short-acting benzodiazepine with a shorter elimination half-life and faster recovery time than midazolam, studies evaluating its safety and efficacy during bronchoscopy are limited. This study aimed to compare the safety and efficacy of remimazolam with those of midazolam for bronchoscopy. This prospective randomized parallel-group study was conducted at a single institution. The primary outcome was the time from the end of the procedure to full alertness. Other procedural time parameters, satisfaction profiles, and adverse effects were thoroughly evaluated. The time taken to reach peak sedation and the time from the end of the procedure to full alertness was significantly shorter in the remimazolam group than in the midazolam group (median [interquartile range], 2 min [1–4] vs. 3 min [2–5], *P* = 0.006; and median, 2 min [1–5] vs. 5 min [1–12], *P* = 0.035, respectively). In patients with non-biopsy procedures (n = 79), participant satisfaction was significantly higher in the remimazolam group than in the midazolam group (median rated scale, 10 vs. 7, *P* = 0.042). Physician satisfaction and willingness to repeat the procedure were similar between groups. Although the incidence of adverse effects was similar between the groups and there was no significant difference, the midazolam group had a higher antidote administration rate than the remimazolam group (15.7% vs. 4.1%, *P* = 0.092). Remimazolam is effective and safe for achieving adequate sedation, with a shorter onset time and faster neuropsychiatric recovery than midazolam. It may be a new option for sedation during bronchoscopy.

**Trial registration**: The trial registration number is NCT05994547, and the date of first registration is 16/08/2023.

## Introduction

Flexible bronchoscopy is important for the diagnosis and treatment of various respiratory diseases^1^. It can be performed without general anesthesia and has the advantage of being safe and fast^2–4^. However, appropriate local anesthesia and sedation are required to perform bronchoscopy successfully.

Local anesthesia can be used to suppress the cough reflex in the airway to facilitate bronchoscopy^5^. Adequate sedation reduces patient discomfort and irritability making it easier to perform the procedure. Therefore, the British Thoracic Society and American College of Chest Physicians recommend performing bronchoscopy under sedation to minimize patient discomfort and complications and increase physician satisfaction^3,4^. Among sedatives, benzodiazepines, especially midazolam, are the most commonly used for bronchoscopy^2,6^. Midazolam is water soluble, has high fat solubility, is painless when injected intravenously, and is rapidly distributed in the central nervous system^7^. However, its long half-life leads to the late recovery of neuropsychiatric function^8,9^. Remimazolam is an ultra-short-acting benzodiazepine that induces sedation by acting on GABA receptors in the same manner as midazolam^10^ but it has a shorter elimination half-life^11^ and has shown a faster recovery time during endoscopy in previous clinical trials^12,13^. Another study compared the safety and efficacy of remimazolam and midazolam in bronchoscopy^14^; however, because both groups used fentanyl, there was no direct comparison between the two drugs.

Therefore, this study aimed to compare the safety and efficacy of remimazolam with those of midazolam for bronchoscopy.

## Results

### Baseline characteristics

The baseline patient characteristics are shown in Table 1. The median age was 67 years (interquartile range [IQR], 57–75), 61% were male, and 59% were ex- or current smokers. Fifty-one patients were assigned to the midazolam group and 49 to the remimazolam group. The median body mass index (BMI) was significantly higher in the remimazolam group than in the midazolam group (23.9 kg/m^2^ vs. 21.9 kg/m^2^, *P* = 0.033). Age, sex, smoking history, and comorbidities were similar between the two groups. The subgroup of patients who underwent non-biopsy procedures (observation alone, saline washing, or secretion suction) was defined as the non-biopsy group (n = 79), whereas the patients who underwent biopsy-containing procedures (endobronchial forceps biopsy with or without saline washing) were defined as the biopsy group (n = 21). The use of midazolam or remimazolam was similar between the subgroups.

**Table 1:** Baseline characteristics.

	Total (N = 100)	Midazolam (n = 51)	Remimazolam (n = 49)	*P*-value
Age, years	67 (57–75)	68 (60–75)	65 (55–74)	0.216
Sex, males	61 (61.0)	30 (58.8)	31 (63.3)	0.649
BMI, kg/m^2^	23.1 (20.4–24.9)	21.9 (19.8–24.0)	23.9 (21.0–25.5)	0.033
Smoking history				
Never smoker	41 (41.0)	21 (41.2)	20 (40.8)	0.717
Ex-smoker	25 (25.0)	11 (21.6)	14 (28.6)	
Current smoker	34 (34.0)	19 (37.3)	15 (30.6)	
Comorbidities				
COPD	10 (10.0)	6 (11.8)	4 (8.2)	0.741
Asthma	4 (4.0)	2 (3.9)	2 (4.1)	1.000
Pulmonary tuberculosis	18 (18.0)	10 (19.6)	8 (16.3)	0.669
Interstitial lung disease	2 (2.0)	0	2 (4.1)	0.238
Diabetes mellitus	20 (20.0)	13 (25.5)	7 (14.3)	0.161
Hypertension	33 (33.0)	17 (33.3)	16 (21.7)	0.942
Cardiovascular disease	12 (12.0)	5 (9.8)	7 (14.3)	0.550
Neurologic disease	3 (3.0)	2 (3.9)	1 (2.0)	1.000
Chronic kidney disease	1 (1.0)	1 (2.0)	0	1.000
Chronic liver disease	1 (1.0)	0	1 (2.0)	0.490
Malignancy	13 (13.0)	6 (11.8)	7 (14.3)	0.708
Subgroup according to procedures*				
Non-biopsy group	79 (79.0)	38 (74.5)	41 (83.7)	0.329
Biopsy group	21 (21.0)	13 (25.5)	8 (16.3)	

Data are presented as medians (interquartile ranges) or numbers (%).

*BMI* body mass index, *COPD* chronic obstructive pulmonary disease.

*Patients who underwent non-biopsy procedures (observation alone, saline washing, or secretion suction) were defined as the non-biopsy group, whereas patients who underwent biopsy-containing procedure (endobronchial forceps biopsy with or without saline washing) were defined as the biopsy group.

### Procedure medication and time

The same dose of instilled 1% lidocaine (150 mg) was used in all patients (Table 2). The total dose of sedative drugs for remimazolam and midazolam was 5 (3–5.5) mg and 3 (2–3) mg, respectively. The initial dose of sedative drugs for remimazolam and midazolam was 2 (2–2) mg and 3 (3–5) mg, respectively. Although there was no statistical significance, the midazolam group had more patients requiring additional drug administration than the remimazolam group (47.1% vs. 32.7%, *P* = 0.142) before procedure initiation. None of the patients required more than two additional drugs. The numbers of patients requiring additional drug administration during the procedure were similar between the two groups (11.8% vs. 12.2%, *P* = 0.941).

**Table 2:** Procedure medication and time.

	Midazolam (n = 51)	Remimazolam (n = 49)	*P*-value
Procedure medication			
1% Lidocaine, mg	150 (150–150)	150 (150–180)	0.287
Total dose of sedative drugs (mg)	3 (2–3)	5 (3–5.5)	
Initial dose of sedative drugs (mg)	2 (2–2)	3 (3–5)	
Presence of additional sedative drug administration	24 (47.1)	16 (32.7)	0.142
Presence of additional sedative drug administration during bronchoscopy	6 (11.8)	6 (12.2)	0.941
Procedure time			
Time taken to reach peak sedation, min	3 (2–5)	2 (1–4)	0.006
Duration of bronchoscopy time, min	4 (3–8)	4 (3–7)	0.796
Time from first dose to end of procedure, min	8 (6–12)	7 (5–10)	0.102
Time from end of procedure to full alertness, min	5 (1–12)	2 (1–5)	0.035

Data are presented as medians (interquartile ranges) or numbers (%).

In terms of procedure time, the time taken to reach peak sedation was significantly shorter in the remimazolam group than in the midazolam group (median time [IQR], 2 (1–4) min vs. 3 (2–5) min, *P* = 0.006). Additionally, the time from the end of the procedure to full alertness (i.e., primary outcome) was significantly shorter in the remimazolam group than in the midazolam group (2 (1–5) min vs. 5 (1–12) min, *P* = 0.035). The time from the first dose to the end of the procedure and the duration of bronchoscopy were similar between the two groups.

### Procedure-related complications and hemodynamic/respiratory parameters

Procedure-related complications were monitored between procedure initiation (drug administration) and hospital discharge. The most frequent procedure-related adverse effect was the need for antidote administration (10%), followed by tachycardia (4%), hypotension (2%), and hypoxemia (2%). The midazolam group showed a higher rate of antidote administration than the remimazolam group, although the difference was not statistically significant (15.7% vs. 4.1%, *P* = 0.092). Hypotension, hypoxemia, and tachycardia were similar between the two groups (Table 3).

**Table 3:** Incidence of adverse events after bronchoscopy.

Adverse events after bronchoscopy	Total (N = 100)	Midazolam (n = 51)	Remimazolam (n = 49)	*P*-value
Bradycardia	0 (0.0)	0 (0.0)	0 (0.0)	
Tachycardia	4 (4.0)	2 (3.9)	2 (4.1)	1.000
Vomiting	0 (0.0)	0 (0.0)	0 (0.0)	
Hypotension	2 (2.0)	1 (2.0)	1 (2.0)	1.000
Hypoxemia	2 (2.0)	1 (2.0)	1 (2.0)	1.000
Use of Antidote	10 (10.0)	8 (15.7)	2 (4.1)	0.092
Pneumonia	0 (0.0)	0 (0.0)	0 (0.0)	
Pneumothorax	0 (0.0)	0 (0.0)	0 (0.0)	
ARDS or ICU admission	1 (1.0)	1 (2.0)*	0 (0.0)	1.000

Data are presented as numbers (%).

*ARDS* acute respiratory distress syndrome, *ICU* intensive care unit.

*One patient (male, 69 years) in the midazolam group developed severe ARDS 24 h after the procedure. He was admitted with anorexia, cough, night sweating, and general weakness for 20 days. Chest computed tomography scan showed multifocal consolidations, and bronchoscopic saline washing was performed. He died 53 h after bronchoscopy owing to aggravated ARDS and multi-organ failure.

Hemodynamic and respiratory parameters were measured at baseline, initiation of the procedure, end of the procedure, and 5 and 10 min after the initiation of the procedure. Systolic and diastolic blood pressure, heart rate, respiratory rate, and oxygen saturation measured using pulse oximetry did not differ between the two groups (Supplemental Table 1).

### Satisfaction profiles

**Figure 1:**
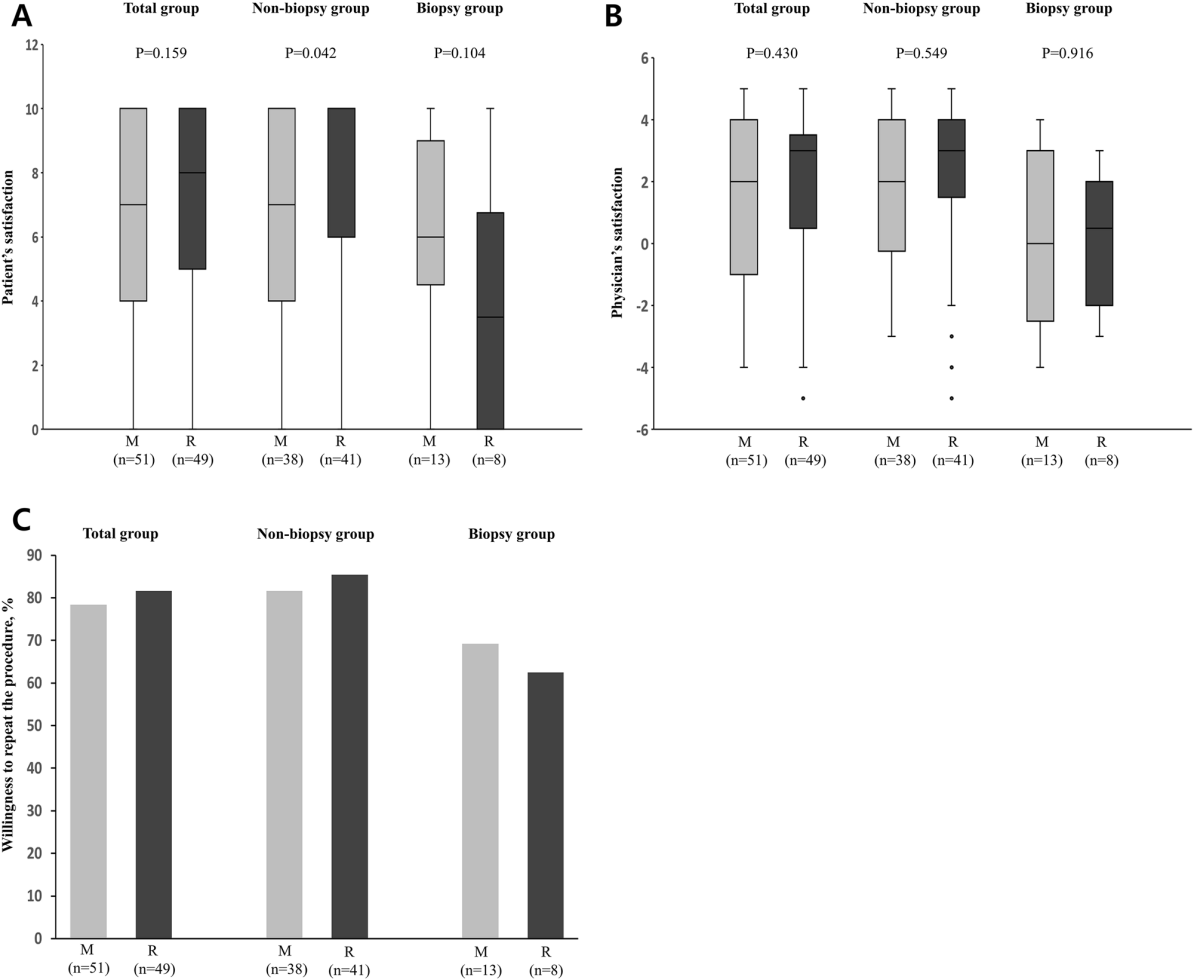
Satisfaction profiles. Box plot of patient and physician satisfaction with sedation ((**A**) and (**B**)). In the box plots, a horizontal black line denotes the median value. Upper and lower boundary of the box denote 1st and 3rd quartile, respectively. Vertical lines above and below the box indicate the 10th and 90th percentiles, respectively. Dots below the vertical line denote outliers outside the 90th percentile. In patients with non-biopsy procedures (n = 79), patient satisfaction was significantly higher in the remimazolam group than in the midazolam group (median [IQR], 10 [6–10] vs. 7 [4–10], *P* = 0.042]. Percentage of patients who showed willingness to repeat the procedure in each group (**C**). *IQR* interquartile range, *M* midazolam, *R* remimazolam.

The degree of patient and physician satisfaction with the procedure was similar between the two groups (Fig. 1). Approximately 78.4% and 81.6% of the midazolam and remimazolam groups, respectively, responded that they were satisfied with the procedure and would be willing to undergo repeat procedures if necessary. In patients with non-biopsy procedures (n = 79), participant satisfaction was significantly higher in the remimazolam group than in the midazolam group (median rated scale, 10 vs. 7, *P* = 0.042). However, physician satisfaction and willingness to repeat the procedure were similar between the two groups. In patients who underwent biopsy (n = 21), patient and physician satisfaction were similar between the two groups. However, 69.2% of the midazolam group and 62.5% of the remimazolam group showed willingness to undergo a repeat procedure.

## Discussion

In this study, we compared the efficacy, safety, and satisfaction profiles of remimazolam and midazolam as sedatives for flexible bronchoscopy. Remimazolam showed a shorter time from the end of the procedure to full alertness and time taken to reach peak sedation than midazolam. Not only were the drug-related side effects acceptable and easily manageable, but patient satisfaction was also significantly higher in the remimazolam group than in the midazolam group, especially for non-biopsy procedures. In addition, although not statistically significant, the remimazolam group tended to require lower antidote (i.e., flumazenil) usage.

The major findings of our study were the rapid sedation and wakefulness induced by remimazolam. These results are consistent with those of a recently published clinical trial by Pastis et al., in which three groups (remimazolam or placebo in a double-blind manner and open-label midazolam) were compared during flexible bronchoscopy^14^. Although a predetermined dose of fentanyl was also administered, remimazolam achieved a higher rate of procedural success and a lower rate of additional sedative medication requirements than midazolam or the placebo. In addition, the mean time for recovery (i.e., the time from the end of the procedure to full alertness) was shorter in the remimazolam group than in the midazolam group. These results correlate with the pharmacokinetic advantages of remimazolam. As remimazolam is rapidly converted into an inactive metabolite, it is characterized by rapid onset and recovery from sedation. Compared with midazolam, remimazolam has a shorter onset time (1–2 min vs. 3–5 min) and recovery time (10–40 min vs. 20–80 min)^15^.

Another notable finding of our study was that remimazolam was associated with higher patient satisfaction than midazolam in a subgroup of patients who underwent non-biopsy procedures. More than 80% of patients in the remimazolam group responded that they were willing to undergo repeat procedures. Several factors affect patient satisfaction after flexible bronchoscopy. Lower levels of anxiety before the procedure, shorter examination time, higher physician quality, and lower levels of discomfort during the procedure are associated with higher patient satisfaction^16–18^. In the present study, the examination time (i.e., the time from the first dose to the end of the procedure) was generally short (less than 10 min). Hence, the pharmacokinetic and pharmacodynamic properties of remimazolam may render non-biopsy procedures with shorter examination times more comfortable. In contrast, patients who underwent biopsy showed lower satisfaction with remimazolam, although the difference was not statistically significant. This can be explained by the lack of synergistic effects of the combination of sedatives and analgesics (such as fentanyl and remifentanil) in the present study. Therefore, we suggest that the combined administration of analgesics and sedatives during bronchoscopy for biopsy could increase patient satisfaction.

To date, many known drugs are used in bronchoscopy procedures. Benzodiazepines, alone or in combination with opioids, are the preferred sedative drugs for flexible bronchoscopy^19^. Various sedative regimens other than benzodiazepines (such as propofol and dexmedetomidine) have also been widely used, and each has unique advantages. Although propofol is usually recommended in an experienced setting because of the narrow therapeutic window and unavailability of antidote^20^, it showed a shorter recovery time and comparable safety profiles compared with midazolam^21,22^. Dexmedetomidine is a highly selective α_2_-agonist that shows both analgesic and sedative effects^23^. Compared with midazolam, it showed better oxygen saturation and comparable patient tolerability^24^. When used as a combination regimen, dexmedetomidine showed better sedative effectiveness, higher bronchoscopist satisfaction, and shorter time to ambulation than midazolam^25^. Remimazolam, as used in our study, may also be an option for use during bronchoscopy.

Remimazolam is similar to midazolam in terms of safety. In addition, remimazolam requires less antidote use after completion because it has the advantage of rapid recovery of neurological function. Benzodiazepines can act on the cardiovascular system causing hypotension and tachycardia and can also cause apnea and hypoxia by suppressing the respiratory center^10,26^. Remimazolam rapidly hydrolyzes ester linkages to produce inactive metabolites, a mechanism that allows minimal residual sedation^10,27^. This mechanism is considered an important advantage, as it increases the safety of remimazolam and requires less antidote administration.

This study has several important clinical implications. Our findings suggest that remimazolam can achieve effective sedation and can be safely administered during bronchoscopy. This effect is also significant because the study directly compared midazolam and remimazolam, while excluding other analgesics (such as fentanyl or remifentanil), whereas, to date, midazolam has been used in combination with fentanyl.

Despite these strengths, this study has several limitations. First, it was confined to a single center, and the sample size was relatively small. However, because the sample size that satisfied the primary endpoint was calculated based on previous studies^14^, it was not expected to affect the results of the study. Second, this was not a completely randomized controlled study. As an example, BMI differed unexpectedly between the midazolam and remimazolam groups. However, patients and physicians were completely unaware of the sedative drugs; therefore, the bronchoscopic procedure and study results were not affected. Third, the Modified Observer's Alertness/Sedation (MOAA/S) scale used patient responsiveness as an index for sedation, and it is acknowledged that the value may vary depending on the measurer. However, we attempted to overcome this shortcoming by having two trained respiratory nurses evaluate sedation.

## Conclusions

Remimazolam is an effective and safe option for achieving adequate sedation during bronchoscopy, with a shorter onset time and faster neuropsychiatric recovery time than midazolam. Additionally, patient satisfaction was significantly higher in the remimazolam group than in the midazolam group, particularly in the non-biopsy group. Therefore, this drug may provide a new option for sedation during bronchoscopy.

## Methods

### Study design and patients

This prospective, randomized, parallel-group study was conducted at Chungbuk National University Hospital between April 2022 and June 2023. Patients aged ≥ 18 years who required diagnostic or therapeutic bronchoscopy and agreed to participate in the study were enrolled. The exclusion criteria were as follows: (1) American Society of Anesthesiologists physical status classification ≥ 4, (2) BMI < 18.4 kg/m^2^ or > 30.0 kg/m^2^, (3) moderate-to-severe hepatic impairment and lactose intolerance, (4) known sensitivity to benzodiazepines, flumazenil, opioids, or naloxone, (5) a history of tracheostomy, (6) SpO_2_ ≤ 90% in ambient air or with no more than 5L/min of O_2_ support, (7) poor patient cooperation, and (8) patients who, by the judgment of the investigator, were not appropriate for the study.

**Figure 2:**
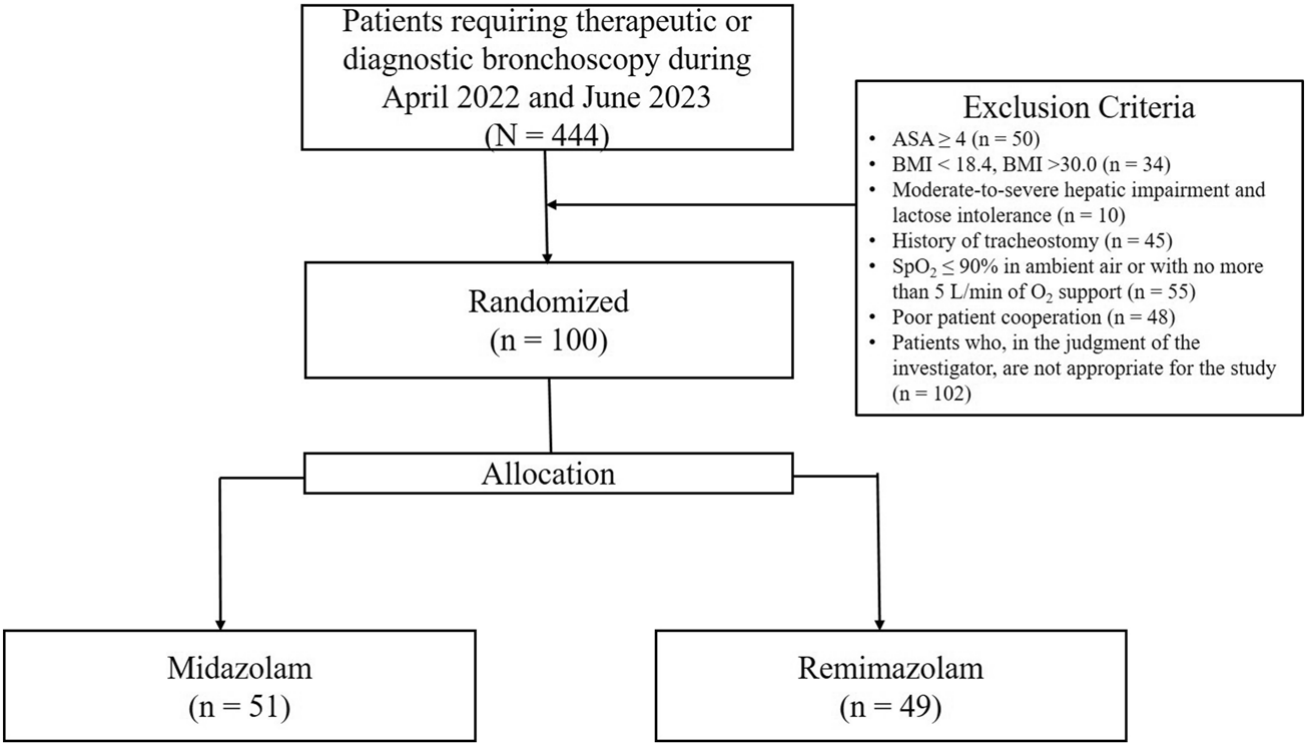
Study population. *ASA* American Society of Anesthesiologists, *BMI* body mass index; *SpO*_*2*_ oxygen saturation.

The patients were randomly assigned to the midazolam or remimazolam group in a 1:1 ratio. Neither the bronchoscopists nor the patients were allowed to know which group they belonged to. Randomization was performed by respiratory nurse specialists in the bronchoscopy unit. Finally, 100 patients were enrolled, of whom 51 were randomly assigned to the midazolam group and 49 to the remimazolam group (Fig. 2).

### Topical anesthesia

Oral and laryngeal anesthesia was induced using a 4% lidocaine nebulizer prior to sedation. The vocal cords and lower airway tract were anesthetized with 3 mL of a 1% lidocaine HCl solution (30 mg of lidocaine) using the spray-as-you-go technique^4^: twice on the vocal cords and once on the trachea and both main bronchi. Additional topical anesthesia was administered at the discretion of the physician.

### Sedation protocol

Patients aged < 60 years or weighing > 50 kg were randomly assigned to receive 3 mg intravenous midazolam or 5 mg remimazolam. In contrast, those aged ≥ 60 years or with a body weight of < 50 kg were assigned to receive 2 mg intravenous midazolam or 3 mg intravenous remimazolam^3,12^. The degree of sedation was measured using the MOAA/S, and bronchoscopy was performed under moderate sedation (MOAA/S = 3)^3,28^. If moderate sedation was not achieved after the initial sedative administration, additional midazolam (0.5 mg) or remimazolam (2.5 mg) was administered at intervals of 3–4 min, at the discretion of the physician.

During bronchoscopy, all of the patients received supplemental oxygen via a nasal cannula at 5 L/min. If oxygen saturation measured by pulse oximetry was < 90%, jaw thrust was performed and the oxygen flow was increased to 10 L/min. Hemodynamic parameters, including continuous electrocardiography, heart rate, respiratory rate, and automated noninvasive blood pressure measurements, were recorded every 5 min.

### Definitions

The time taken to reach peak sedation was defined as the time to adequate sedation (MOAA/S = 3). The time taken to reach peak sedation was defined as the time to the start of the procedure after the administration of the first dose of medication. The duration of bronchoscopy was defined as the period from bronchoscope insertion to mouth exit. The time from the first dose to the end of the procedure was defined as the time from the first sedative drug administration to mouth exit. The time from the end of the procedure to when the patient was fully alert was defined as when MOAA/S had recovered to 5 after the procedure had been completed.

### Outcomes

The primary outcome was the time from the end of the procedure to full alertness in both groups. We further analyzed the time taken to reach peak sedation and from administration of the first dose to the end of the procedure.

The secondary outcomes were physician and patient satisfaction after the procedure. Patient satisfaction was measured using a visual analog scale (0 = intolerable, 10 = free from inconvenience). After the patients had fully recovered, we asked them about their willingness to repeat the procedure. Physician satisfaction with sedation was expressed as a score ranging from − 5 to 5 after completion of the procedure (− 5 = not satisfied at all, 5 = maximally satisfied). Additionally, we investigated whether there was a difference between the adverse effects that occurred after bronchoscopy in the midazolam and remimazolam groups.

### Statistical analysis

Based on previous studies, the sample size was calculated by performing a two-sample t-test with a power of 80% and a significance level of 5%. Considering a drop-out rate of 20%, 73 patients were required, and 100 patients were enrolled in this study^14^.

Data are presented as median and IQR for continuous variables and as frequency (percentage) for categorical variables. Continuous variables were compared using the Mann–Whitney U test, and the Pearson chi-squared test or Fisher’s exact test was used for categorical variables. All tests were two-sided, and *P*-values < 0.05 were considered statistically significant. All statistical analyses were performed using the IBM SPSS Statistics for Windows software (version 27.0; IBM Corp., Armonk, NY, USA).

### Ethics approval

The study protocol was reviewed and approved by the Institutional Review Board of the Chungbuk National University Hospital (IRB No 2022-03-002). The study was conducted in accordance with the principles of the Declaration of Helsinki. Written informed consent was obtained from all patients on enrollment. When obtaining informed consent, baseline demographic characteristics, comorbidities, and the purpose of bronchoscopy were investigated.

### Supplementary Information

Supplementary Table 1.

## Data Availability

The data presented in this study are available on request from the corresponding author.
