# Supplementary material for: Safety and efficacy of remimazolam compared with midazolam during bronchoscopy: a single-center, randomized controlled study
Source: Sci Rep. 2023 Nov 22;13:20498. doi: 10.1038/s41598-023-47271-w (PMC10665376; doi:10.1038/s41598-023-47271-w)
Supplement: Supplementary file 1 — Supplementary Table 1. [file 41598_2023_47271_MOESM1_ESM.docx]

**Supplemental table 1.** Hemodynamic and Respiratory parameter during Bronchoscopy

|  | **Midazolam**  **(n = 51)** | **Remimazolam**  **(n = 49)** | ***P*-value** |
| --- | --- | --- | --- |
| Initial | n = 51 | n = 49 |  |
| SBP | 134 (125-156) | 138 (125-157) | 0.879 |
| DBP | 83 (77-90) | 84 (94-77) | 0.469 |
| Pulse | 76 (70-88) | 83 (70-88) | 0.684 |
| RR | 16 (15-19) | 16 (13-20) | 0.392 |
| SpO_2_ | 100 (100-100) | 100 (100-100) | 0.418 |
| Start bronchoscopy | n = 51 | n = 49 |  |
| SBP | 146 (127-173) | 145 (124-171) | 0.509 |
| DBP | 91 (114-81) | 95 (80-114) | 0.957 |
| Pulse | 103 (88-116) | 97 (84-107) | 0.185 |
| RR | 21 (16-24) | 20 (17-25) | 0.994 |
| SpO_2_ | 100 (99-100) | 100 (98-100) | 0.276 |
| 5 minutes after the start of the procedure | n = 21 | n = 23 |  |
| SBP | 163 (143-180) | 153 (122-165) | 0.097 |
| DBP | 91 (78-104) | 91 (82-98) | 0.860 |
| Pulse | 100 (88-110) | 97 (83-102) | 0.254 |
| RR | 21 (18-25) | 20 (16-24) | 0.525 |
| SpO_2_ | 97 (96-100) | 98 (96-99) | 0.694 |
| 10 minutes after the start of the procedure | n = 9 | n = 5 |  |
| SBP | 153 (133-175) | 163 (137-184) | 0.789 |
| DBP | 91 (83-104) | 84 (79-126) | 0.894 |
| Pulse | 103 (91-112) | 99 (93-107) | 0.738 |
| RR | 19 (16-25) | 22 (22-25) | 0.203 |
| SpO_2_ | 97 (94-98) | 96 (96-97) | 0.786 |
| 15 minutes after the start of the procedure | n = 1 | n = 1 |  |
| SBP | 145 (145-145) | 154 (154-154) | 0.317 |
| DBP | 81 (81-81) | 99 (99-99) | 0.317 |
| Pulse | 99 (99-99) | 87 (87-87) | 0.317 |
| RR | 23 (23-23) | 27 (27-27) | 0.317 |
| SpO_2_ | 100 (100-100) | 97 (97-97) | 0.317 |
| At the end of the procedure | n = 51 | n = 49 |  |
| SBP | 150 (134-168) | 144 (123-164) | 0.291 |
| DBP | 85 (76-96) | 86 (82-96) | 0.661 |
| Pulse | 97 (85-107) | 93 (84-106) | 0.460 |
| RR | 21 (18-25) | 21 (16-24) | 0.310 |
| SpO_2_ | 99 (96-100) | 99 (97-100) | 0.851 |

Data are presented as medians (interquartile ranges).

Abbreviations: SBP, systolic blood pressure; DBP, diastolic blood pressure; RR, Respiratory rate; SpO_2,_ Oxygen saturation
